# HLA and Histo-Blood Group Antigen Expression in Human Pluripotent Stem Cells and their Derivatives

**DOI:** 10.1038/s41598-017-12231-8

**Published:** 2017-10-12

**Authors:** Karin Säljö, Angela Barone, Johan Mölne, Lennart Rydberg, Susann Teneberg, Michael E. Breimer

**Affiliations:** 1 Department of Surgery, Institute of Clinical Sciences, Sahlgrenska Academy at University of Gothenburg, Sahlgrenska University Hospital, Gothenburg, Sweden; 2 Department of Medical Biochemistry and Cell Biology, Institute of Biomedicine, Sahlgrenska Academy at University of Gothenburg, Gothenburg, Sweden; 3 Department of Pathology and Genetics, Institute of Biomedicine, Sahlgrenska Academy at University of Gothenburg, Sahlgrenska University Hospital, Gothenburg, Sweden; 4 Department of Clinical Immunology and Transfusion Medicine, Institute of Biomedicine, Sahlgrenska Academy at University of Gothenburg, Sahlgrenska University Hospital, Gothenburg, Sweden

## Abstract

One prerequisite for a successful clinical outcome of human pluripotent stem cell (hPSC) based therapies is immune compatibility between grafted cells/tissue and recipient. This study explores immune determinants of human embryonic stem cell lines (hESC) and induced human pluripotent stem cell (hiPSC) lines and hepatocyte- and cardiomyocyte-like cells derived from these cells. HLA class I was expressed on all pluripotent hPSC lines which upon differentiation into hepatocyte-like cells was considerably reduced in contrast to cardiomyocyte-like cells which retained class I antigens. No HLA class II antigens were found in the pluripotent or differentiated cells. Histo-blood group carbohydrate antigens SSEA-3/SSEA-4/SSEA-5, Globo H, A, Le^x^/Le^y^ and sialyl-lactotetra were expressed on all hPSC lines. Blood group AB(O)H antigen expression was in accordance with *ABO* genotype. Interestingly, only a subpopulation of *A1O1* cells expressed A. During differentiation of hPSC, some histo-blood group antigens showed congruent alteration patterns while expression of other antigens differed between the cell lines. No systematic difference in the hPSC cell surface tissue antigen expression was detected. In conclusion, hPSC and their derivatives express cell surface antigens that may cause an immune rejection. Furthermore, tissue antigen expression must be established for each individual stem cell line prior to clinical application.

## Introduction

The potential clinical applications of stem cell-based technology and products, derived from human embryonic stem cells (hESC) isolated from the inner cell mass of blastocysts^1^ and human induced pluripotent stem cells (hiPSC) generated from adult cells^2^, are currently explored. Human pluripotent stem cells (hPSC, i.e. hESC and hiPSC) can under optimal culturing conditions be propagated indefinitely while maintaining their ability to differentiate into all human cell types^3^. Besides being an endless source of material for tissue engineering and replacement therapy^4^, both the pluripotent and the differentiated cell states can serve as *in vitro* models of various human diseases as well as disease-free controls, thereby facilitating drug development and toxicology screening.

One of the barriers to overcome before hPSC-derived products can be brought to the clinic is the challenge of the recipient’s immune system to non-self antigens, which may command for lifelong immunosuppressive therapy. Generating and maintaining patient-specific hiPSC lines, which may overcome this obstacle^5–7^, is at present costly and time consuming. However, a more feasible approach to enable non-autologous therapies may be to assemble hPSC line banks with diverse HLA (human leukocyte antigen) and ABO blood group types.

Initially hPSC were assumed to be immune privileged due to their undifferentiated state, which partly was reinforced by early experimental data^8^. However, several studies have contradicted this assumption^9,10^. Seemingly, hPSC and their derivatives are subject to the same immunological barriers as conventional allografts. The strongest histocompatibility antigen barriers are the HLA antigens and the ABO blood group systems. HLA class I (HLA-A/B/C) antigens are expressed on almost all nucleated cells^11^, while class II (HLA-DR/DQ/DP) antigens are constitutively expressed mainly on antigen presenting cells but can be induced by cytokines, mainly interferon-gamma. Early studies of hESC demonstrated low levels of HLA class I antigens, with a modest induction during differentiation, and absence of HLA class II^12,13^. Similar results have been reported for hiPSC^14^. In a recent study, including both hESC and hiPSC lines, Chen *et al*. found normal and varying HLA haplotypes as well as confirmatory phenotypic expression of low levels of HLA class I^15^.

Adult cardiomyocytes do not express HLA class I or II, but class I can be found in patients with myocarditis or allograft rejection^16,17^. In contrast, the presence of HLA class I antigens on hepatocytes is not clearly established. Several studies failed to detect HLA class I in normal livers^18–23^, while other reported weak or variable expression^15,24–26^. Adult hepatocytes do not express HLA class II, although expression is inducible in patients with immune mediated liver disease^24,25,27^.

Regarding the ABO blood group system it was initially unclear whether hPSC and their derivatives express AB(O)H antigens^28^. Mölne *et al*. demonstrated that several hESC lines expressed A/B antigens corresponding to the *ABO* genotype^29^. During differentiation into cardiomyocyte-like cells, A/B antigen expression was lost while the antigens were retained in hepatocyte-like cells. AB(O)H antigens are not present in adult cardiomyocytes^30^ or hepatocytes^27,31^.

Several stage-specific antigens (SSEA) of carbohydrate nature have been identified in mice during early embryo development^32,33^. Studies of AB(O)H and Lewis blood group antigen expression during human embryonic development are few. However, Szulman was able to study different fetal tissues and organs from fetuses 5–15 weeks of gestational age and found an inverse correlation between age and distribution^34–36^. Certain tissues showed a consecutive expression during development, while others demonstrated a diminishing trend and a few, including liver and heart, lacked AB(O)H antigens within the observed timeframe.

This study explored the phenotype expression of HLA antigens, histo blood group AB(O)H and related carbohydrate antigens in correlation to the individual *HLA* and *ABO* genotypes in three hESC and three hiPSC lines by flow cytometry (FC) and immunohistochemistry (IH). Studies of total glycosphingolipid fractions as well as protein extracts of the cells were performed in an attempt to differentiate determinants carried by lipids or proteins. In addition, we explored the alterations of these antigens during differentiation into cardiomyocyte- and hepatocyte-like cells.

## Materials and Methods

### hESC lines, culture and differentiation procedures

The hESC lines SA121 and SA181 (Takara Bio Europe AB) originate from human *in vitro* fertilized embryos (Sahlgrenska university hospital Sweden). The GMP-grade hESC line Val 9, derived as previously described^37–40^, was obtained from the National Stem Cell Bank of Spain and developed under xeno-free conditions aimed for clinical applications^39^. The hiPSC lines ChiPSC4, ChiPSC15 and ChiPSC22 (Takara Bio Europe AB) were derived from human dermal fibroblasts using retroviral programming^40,41^.

The cells were thawed, maintained, and passaged in the feeder-free Cellartis® DEF-CS™ 500 Culture System (Takara Clontech, Y30010) according to the manufacturer’s recommendations.

The cell lines were used in subsequent differentiation experiments at the following passages: SA121 p.9–13, 15–22, 24; SA181 p.8, 10–18, 21–22, 24–25; Val 9 p 29, 31, 33; ChiPSC4 p.12, 17, 18, 23; ChiPSC15 p.23, 24; ChiPSC22 p.20, 21.

The hPSC were differentiated into definitive endoderm (DE) cells by applying Cellartis® Definitive Endoderm differentiation kit (Takara Clontech, Y30035). At day 7, the cells were harvested according to the manufacturer’s recommendations and differentiated into hepatocyte-like cells applying the Cellartis® Hepatocyte differentiation kit (Takara Clontech, Y30050,^42^). Cells for analysis were harvested as single cells at different time points as described below. The hiPSC line ChiPSC22 was differentiated into cardiomyocyte-like cells according to Takara Bio Europe AB´s standard protocol (Cellartis® Cardiomyocytes, Y10075).

### Genomic HLA and ABO blood group typing

DNA was extracted from cell suspensions using a biorobot (EZ1, Qiagen). HLA-A/B/C, DRB1, DQA1 and DQB1 typing was performed on a Luminex platform (Luminex) using PCR-SSO technique (OneLambda) and Fusion 2.0 software (OneLambda).

ABO-typing was achieved by a modified PCR-SSP method according to a protocol from Downing and Darke^43^, using Taq PCR Master Mix Kit (QIAGEN, 201443) on a GeneAmp PCR System 9700 (Applied Biosystems). Primers were bought from Scandinavian Gene Synthesis and the gels were analyzed using a LumiBIS detection system (DNR Bio-Imaging Systems Ltd).

### Flow Cytometry (FC)

Characterization of the HLA and histo-blood group antigen expression on the cell surface of hPSC lines and alterations during differentiation into hepatocyte-like and cardiomyocyte-like cells was performed using single-color fluorescence. For detailed description of cell preparation, staining methodology and data analysis see Supplemental information text and Figure S1. Briefly, trypsinized single cell suspensions of 2−3 × 10^5^ cells were incubated at 4 °C with primary monoclonal antibodies (Supplemental Table S1), followed by corresponding FITC-conjungated secondary antibodies and isotype controls (Supplemental Table S2) after extensive washing-procedure. Duplicate samples were prepared and all hPSC lines and cells at consecutive differentiation days were examined on three separate occasions. The samples were analyzed by a FACSCalibur^TM^ flow cytometer (Becton Dickinson), with the use of CellQuest^TM^ and (Becton Dickinson) FlowJo (v10.1.r5, FlowJo LLC) software.

### Complement-dependent cytotoxicity crossmatch (CDC-XM)

Functional assays of the HLA A and B series were performed according to the protocol for assessing recipient and donor compatibility prior to organ transplantation^44^. In brief, the hPSC were incubated with HLA-specific sera with known HLA A and B specificity. Thereafter, rabbit complement (Bio-Rad Medical, 824050) and fluorescent dyes (acridine orange and ethidium bromide) were added. The plates were rotated manually, mounted using cover slips and paraffin and incubated for 30 min. The reaction was evaluated using fluorescence microscopy.

### Immunohistochemistry (IH)

Immunohistochemical analyses of HLA and histo-blood group antigen expression on hPSC lines and their alterations during differentiation were performed as previously described^29^. Cells were harvested by trypsin digestion or scraped of tissue culture flasks as monolayers and fixed in buffered paraformaldehyde. After paraffin embedding, and sectioning, antigen retrieval was performed by microwave treatment. Immunostainings were performed either in a computer-assisted Autostainer Plus processor (Dako) or manually using a Vectastain Elite ABC kit (Vector Laboratories/BioNordika), see Supplemental Table S1 for details.

### Isolation and characterization of lipid-linked carbohydrate antigens

Total neutral (non-acid) and acid glycosphingolipid fractions were isolated from 1 × 10^9^ hESC of cell line SA121 and SA181 respectively, and individual glycosphingolipid components were structurally characterized^45–47^. Thin-layer chromatography and chromatogram binding assays were performed using aluminum- or glass-backed silica gel 60 high performance thin-layer chromatography plates eluted with chloroform/methanol/water (60:35:8, v/v/v) as solvent system. Glycosphingolipids were applied to the plates in quantities of 1–4 µg of pure glycosphingolipids and 40–80 µg of total glycosphingolipid fractions. Chemical detection was done with anisaldehyde and monoclonal antibody reactivity with glycosphingolipids was tested as previously described^29,46,47^.

### Characterization of protein-linked antigens

Western blot (WB) protein binding assay was performed as previously described^47^. Briefly, 13 µg of cell lysates from hESC lines SA121 and SA 181 and 1 µg of the neoglycoproteins were diluted, reduced, heated and loaded onto Bis-Tris gels (NuPage). After electrophoresis, the proteins were stained with Imperial^TM^ Protein Stain (Thermo Scientific) or transferred to nitrocellulose membranes (Bio-Rad), blocked for 1 h and thereafter incubated with primary antibodies (Table S1) over night at 4 °C, followed by incubation with alkaline phosphatase-conjugated secondary antibodies (Table S2) for 1 h at room temperature. Nitroblue tetrazolium dye/5-bromo-4-chloroindol-2-yl phosphate (NBT/BCIP, Sigma) was used for visualization. The experiments were repeated three times.

## Results

### Characterization of HLA and histo-blood group antigens in hPSC lines

**Figure 1:**
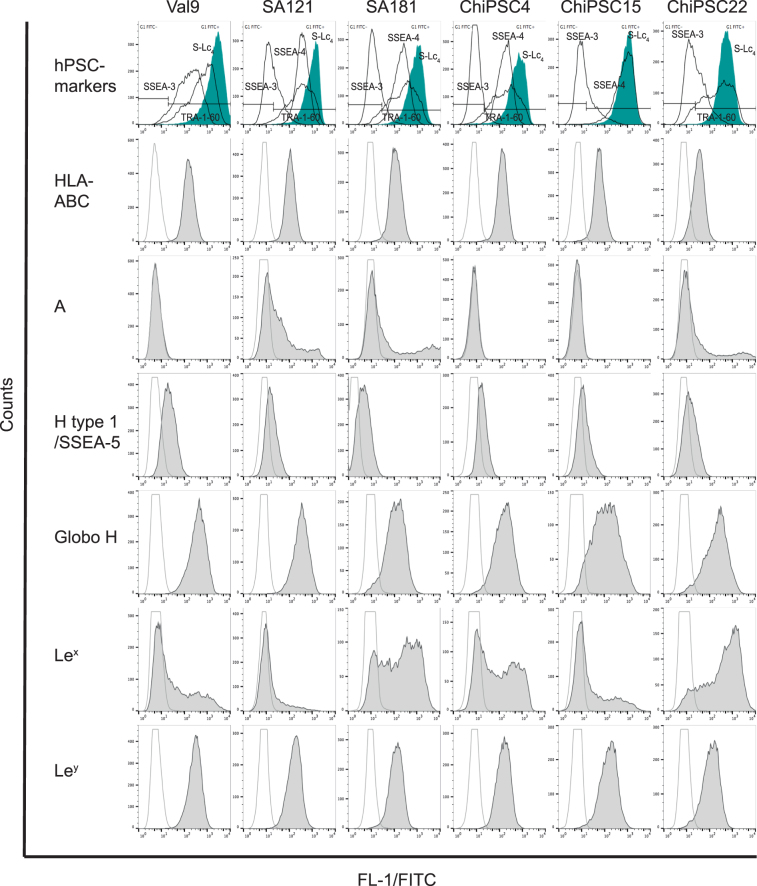
Expression of cell surface HLA and histo-blood group antigens on human pluripotent stem cells analyzed by flow cytometry. Regarding the hPSC markers, black curves represents FITC labeled anti-SSEA-3, anti-SSEA-4 and anti-TRA-1–60 antibodies. The horizontal bars represent FITC negative (FITC−) or positive (FITC+) cells and the gate is set when 99% of the negative control sample falls within the negative gate. The gating-procedure is described in detail under supplemental information and Figure S1. Filled green histograms shows expression of sialyl-lactotetra (S-Lc_4_). FITC labeled anti-HLA and anti-histo-blood group antibodies are shown by filled grey histograms. The transparent grey curves are negative controls, consisting of cells stained exclusively with relevant FITC conjugated secondary antibody (equivalent to corresponding isotype control histogram). In general, all experiments were repeated three times and all samples were duplicated. The figure presents flow cytometry histograms from one representative analysis. *Abbreviations: hPSC, human pluripotent stem cells*; *S-Lc*_4_ *, sialyl-lactotetra*; *A, blood group A*; *Le* ^*x*^ *, Lewis x, Le* ^*y*^ *, Lewis y*.

**Figure 2:**
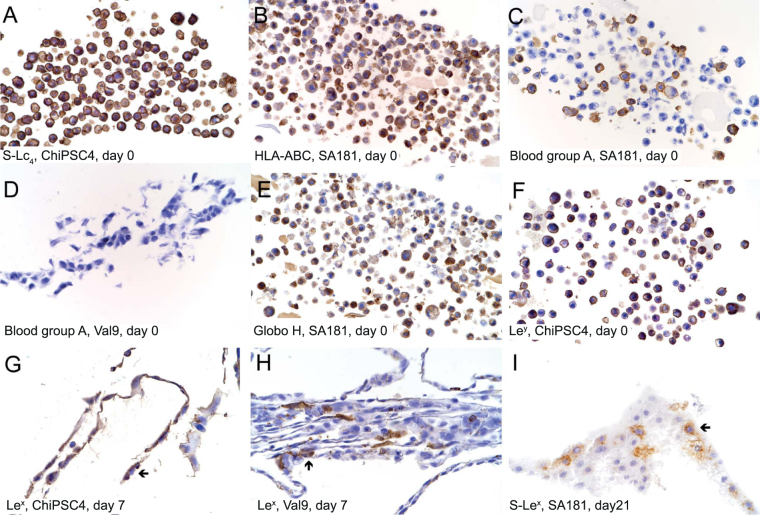
Immunohistochemical analysis of HLA and histo-blood group antigen expression in human pluripotent stem cells and their derivatives. Immunohistochemistry images A–F represents preparations of cells that have been trypsinized into monosuspension, while images G and H illustrate bands of cells that have been manually scraped from the cell culture flask. Immunohistochemical analysis of selected cell lines are presented to illustrate distribution and localization patterns of a specific antigen representative for all cell lines with positive staining with the antibody in question. All pluripotent stem cell lines included in this study express S-Lc_4_ (**A**), HLA-ABC (**B**), Globo H (**E**), and Le^y^ (**F**). Blood group A antigens were expressed on the cell surface of approximately 35% of SA181 cells genotyped as blood group *A*_1_ */0* (**C**), while the blood group O cell line Val 9 was completely negative (**D**). A subpopulation of cells expressed Le^x^ antigens at day 7 of differentiation into hepatocyte-like cells, illustrated by cell line ChiPSC4 (**G**) and Val 9 (**H**). The expression of S-Le^x^ increased during differentiation into hepatocyte-like cells and most evidently in hESC line SA181 (**I**). *Abbreviations: S-Lc*_4_ *, sialyl-lactotetra*; *Le* ^*x*^ *, Lewis x*; *Le* ^*y*^ *, Lewis y*; *S-Le* ^*x*^ *, sialyl-Lewis x*.

**Figure 3:**
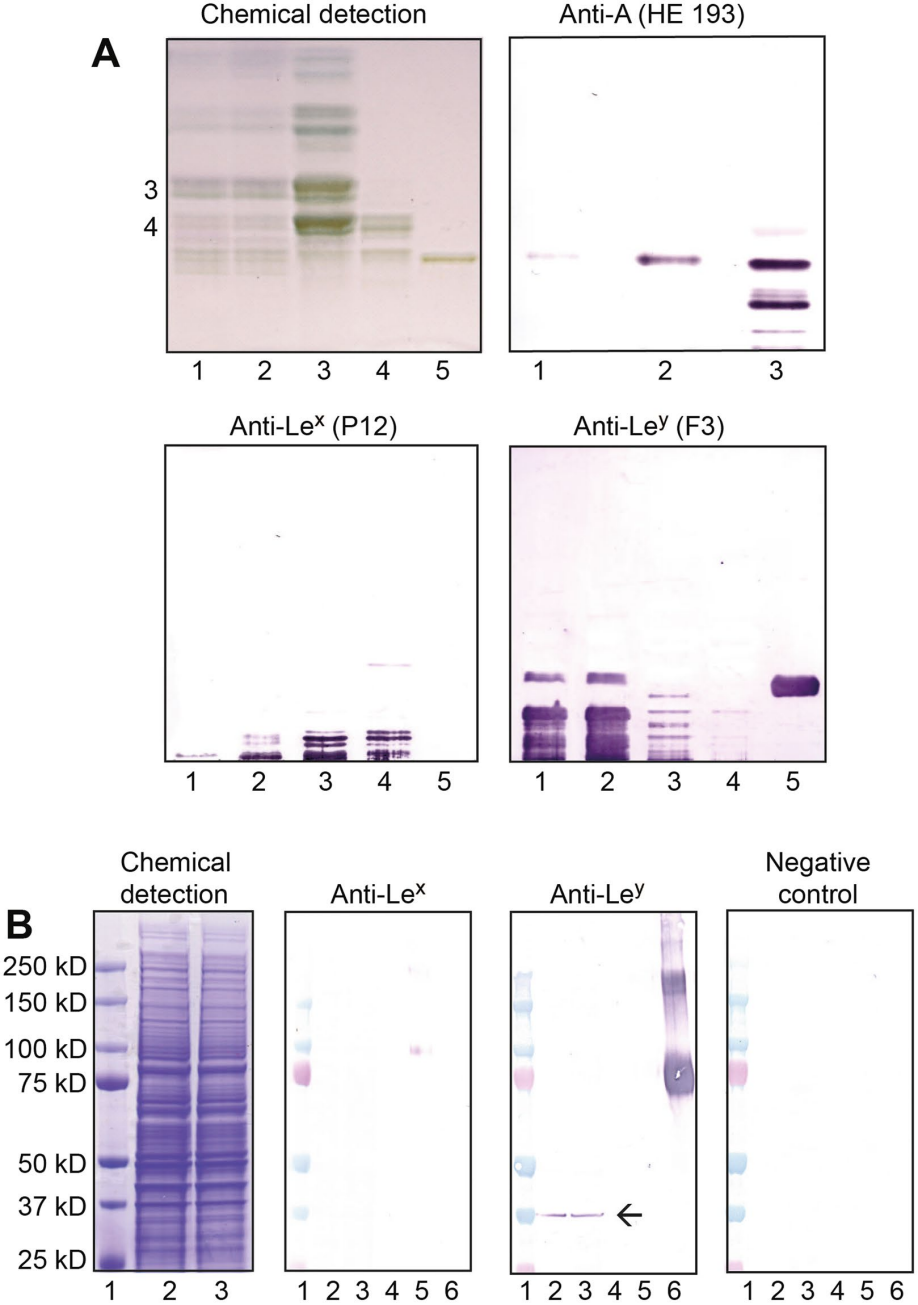
Binding of monoclonal antibodies to total neutral glycosphingolipid fractions (**A**) and protein extracts (**B**) isolated from human embryonic stem cell (hESC) lines SA121 and SA181. Thin-layer chromatograms after detection with chemical anisaldehyde reagent and immunostaining using anti-blood group A, anti-Le^x^ and anti-Le^y^ antibodies are shown in **A**. *Lane 1*, neutral glycosphingolipids of cell line SA121; *lane 2*, neutral glycosphingolipids of cell line SA181; *lane 3*, neutral glycosphingolipids reference fraction from human blood group A_1_ erythrocytes^64^; *lane 4*, neutral glycosphingolipids of human blood group A kidney^65^; *lane 5*, reference Le^y^, Fucα2Galβ4(Fucα3)GlcNAcβ3Galβ4Glcβ1Cer glycosphingolipid. The numbers to the left of the chromatogram denote approximate number of carbohydrate residues in the bands. Western blot analysis of hESC protein extracts separated on 4–12% Bis-Tris gels, immunostained with anti-Le^x^ and anti-Le^y^ monoclonal antibodies are shown in **B**. Chemical staining (Imperial^TM^ protein stain) revealed numerous bands ranging from < 10- >250 kD as indicated to the left). The arrow indicates anti-Le^y^ antibody positive bands. The anti-Le^x^ antibody showed unspecific background staining, with a similar pattern as the negative control with secondary antibody background staining. *Lane 1*, molecular mass standards; *lane 2*, protein extract of hESC line SA121, 13 µg; lane 3, protein extract of hESC line SA181, 13 µg; lane 4, reference H type 2 neoglycoprotein, 1 µg; lane 5, reference Lex neoglycoprotein, 1 µg; lane 6, reference Ley neoglycoprotein, 1 µg. *Abbreviations: A, blood group A; Lex, Lewis x, Ley, Lewis y.*

**Figure 4:**
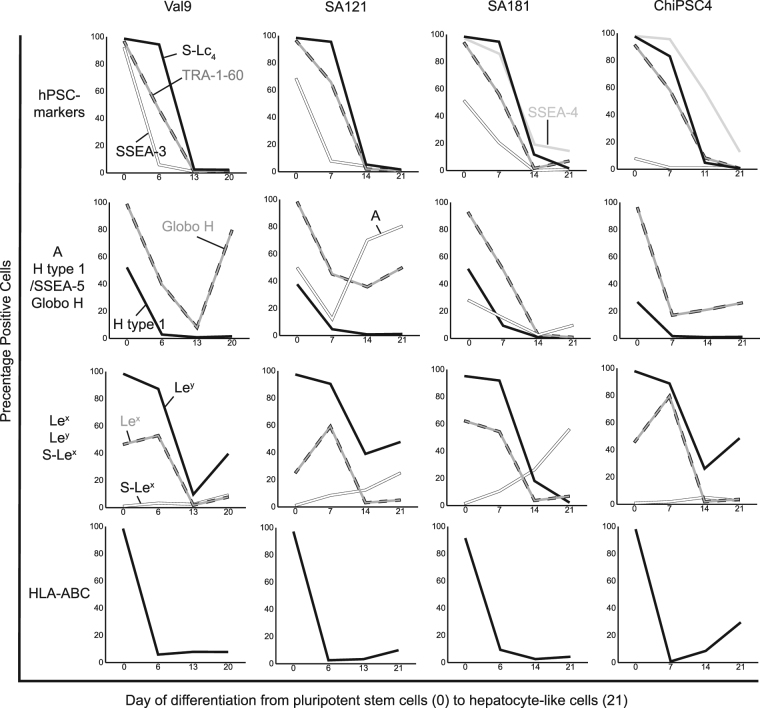
Alteration of HLA and histo-blood group antigen expression during differentiation of human pluripotent stem cells into hepatocyte-like cells. The human embryonic stem cell lines Val 9, SA121 and SA181, and the human induced pluripotent stem cell line ChiPSC4 were characterized by flow cytometry with monoclonal antibodies. Days of differentiation are given on the x-axis and the percentages of cells expressing the different antigens are given on the y-axis. In general, all experiments were repeated three times and all samples were duplicated. The figure presents the percentage of positive cells from one representative analysis. *Abbreviations: hPSC, human pluripotent stem cells*; *S-Lc*_*4*_ *, sialyl-lactotetra*; *A, blood group A*; *Le* ^*x*^ *, Lewis x, Le* ^*y*^ *, Lewis y, S-Le* ^*x*^ *, sialyl-Lewis x*.

**Figure 5:**
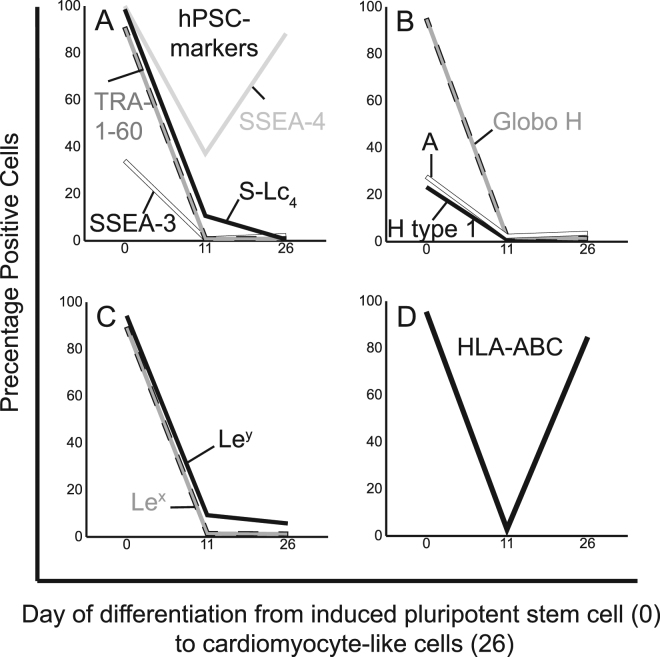
Alteration of antigen expression during differentiation of human induced pluripotent stem cell line ChiPSC22 into cardiomyocyte-like cells. The human induced pluripotent stem cell line ChiPSC22 was differentiated into cardiomyocyte-like cells and the cell surface antigen expression of different HLA and histo-blood group antigens was analyzed. Days of differentiation are given on the x-axis and the percentages of cells expressing the different antigens are given on the y-axis. In general, all experiments were repeated three times and all samples were duplicated. The figure presents the percentage of positive cells from one representative analysis. *Abbreviations*: *hPSC, human pluripotent stem cells*; *S-Lc*_*4*_, *sialyl-lactotetra*; *A, blood group A*; *Le* ^*x*^, *Lewis x, Le* ^*y*^, *Lewis y, S-Le* ^*x*^, *sialyl-Lewis x*.

**Figure 6:**
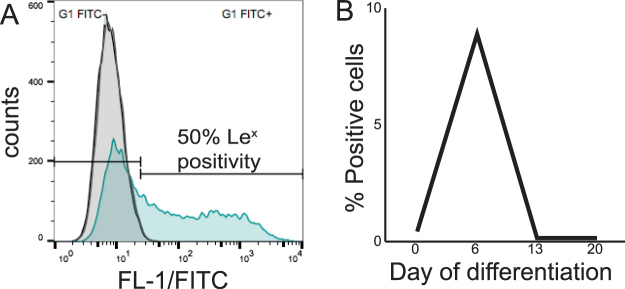
Cell surface staining with antibody P12 (anti-Le^x^) and antibody MC480 (anti-SSEA-1) of human embryonic stem cell line Val 9 and alterations of SSEA-1 expression during differentiation into hepatocyte-like cells. The figure illustrates the expression of the human embryonic stem cell line Val 9 in the pluripotent state (**A**) and during differentiation into hepatocyte-like cells (**B**). (**A)** Representative histogram illustrating the expression on Le^x^ (filled green curve) and SSEA-1 (filled grey curve) which overlaps the negative control (transparent black curve). (**B)** Approximately 10% of the cells transiently expressed SSEA-1 (MC480 antibody) on day 7 of differentiation.

The expression of cell surface HLA and histo-blood group antigens on hESC (Val 9, SA121 and SA181) and hiPSC (ChiPSC4, ChiPSC15 and ChiPSC22) lines was analyzed by flow cytometry (FC) and immunohistochemistry (IH) and the results are shown in Figs 1, 2 and summarized in Table 1. In an attempt to characterize the molecular structure of the antigens, immunostaining of purified glycosphingolipid fractions separated by thin-layer chromatography and western blot (WB) of extracted protein fractions were performed and selected experimental data is shown in Fig. 3. Antigen expression alterations during differentiation into hepatocyte- and cardiomyocyte-like cells are shown in Figs 2, 4, 5, 6B and S3. All cell lines analyzed showed a homogenous staining intensity and antigen expression pattern with no significant variation in antigen expression between different cell passages and experimental repetitions. No major systematic differences were observed between the hESC and hiPSC lines. The chemical structure and short hand designations of the histo-blood group carbohydrate determinants are shown in Table S3.

**Table 1:** Genotype and expression of HLA and histo-blood group antigens in six different human pluripotent stem cell lines.

	*Human Embryonic Stem Cell Lines*			*Human Induced Pluripotent Stem Cell Lines*		
	Val 9	SA121	SA181	ChiPSC4	ChiPSC15	ChiPSC22
Genotyping						
HLA	HLA- A*02,*11 B*07,*51 C*2,*07 DQA1*01,*05 DQB1*03,*06 DRB1*11,*15	HLA- A*32,*68 B*07,*44 C*07;*07 DQA1*01,*01 DQB1*06,*06 DRB1*13,*15	HLA- A*02,*68 B*08,*39 C*07,*12 DQA1*02,*05 DQB1*02,*02 DRB1*03,*07	HLA- A*23,*30 B*53,*58 C*04,*07 DQA1*01,*01 DQB1*05,*06 DRB1*15,*16	HLA- A*02,*29 B*44,*49 C*07,*16 DQA1*02,*03 DQB1*02,*03 DRB1*04,*07	HLA- A*02,*02 B*07,*40 C*03,*07 DQA1*01,*01 DQB1*05,*06 DRB1*13,*14
ABO	*O1O1*	*A1O1*	*A1O1*	*O1O1*	*O1O1*	*A1O1*
Phenotyping^1^						
HLA-ABC	/−^1^	+/	+/+	+/+	+/	+/
HLA-DR/DQ/DP	−/−	−/	−/−	−/−	−/	−/
S-Lc_4_	+/	/+	+/+	+/+	+/	+/
SSEA-4	/	+/+	+/+	+/+	+/	+/
SSEA-3	+/	+/+	+/	/+	+/	/
Globo H	+/-	+/+	+/+	+/+	+/	+/
H type 1/SSEA-5	+/-	+/	+/-	+/−	+/	+/
A	−/−	50% pos/+	35% pos/+	−/−	−/	30% pos/
B	−/	−/−	−/−	−/	−/	−/
SSEA-1	−/−	−/	−/−	−/−	−/	−/
Le^x^	45% pos/-	25% pos/-	65% pos/-	50% pos/-	40% pos/	90% pos/
S-Le^x^	−/−	−/−	−/−	−/−	−/	−/−
Le^y^	+/+	+/+	+/+	+/+	+/	+/
Le^a^	−/	−/	−/	−/	−/	−/
Le^b^	−/	−/	−/	−/	−/	−/
Forssman		−/	−/	−/	−/	−/

^1^Results of Flow cytometry and Immunohistochemistry analysis are given by + (positive) or – (negative) for each analytical method (FC/IH). Absence of + or − indicate that the antibody was not tested with the method in question.

Three human embryonic stem cell lines (Val 9, SA121 and SA181) and three human induced pluripotent stem cell lines (ChiPSC4, ChiPSC15 and ChiPSC22) were studied. The results from HLA and ABO genotyping are presented in the upper part of the table and the results from flow cytometry (FC) and immunohistochemical (IH) phenotyping of the HLA and histo-blood group antigen expression in the cell lines are shown in the lower part of the table. For details regarding primary antibodies used in FC and IH see supplemental Table 1.

*Abbreviations: S-Lc*_4_ *, sialyl-lactotetra*; *A, blood group A*; *B, blood group B*; *Le* ^*x*^ *, Lewis x*; *Le* ^*y*^ *, Lewis y*; *S-Le* ^*x*^ *, sialyl-Lewis x*; *Le* ^*a*^ *, Lewis a*; *Le* ^*b*^ *, Lewis b*.

### Pluripotency markers

The pluripotency state for each cell line was evaluated by phenotypic profiling of established markers such as TRA-1–60, SSEA-3 and SSEA-4 and the recently identified sialyl-lactotetra (S-Lc_4_)^47^. All hPSC lines abundantly expressed these markers (Figs 1 and 2A). However, in contrast to anti-S-Lc_4_, anti-TRA-1–60 and anti-SSEA-4, the FC analysis with anti-SSEA-3 showed a diverse binding between cell lines and only a modest expression. Immunohistochemistry revealed mainly an intracellular expression of SSEA-3 (Figure S2A), whereas the other pluripotency markers in addition were localized on the cell surface, most evident for S-Lc_4_ (Fig. 2A). The H type 1/SSEA-5 antigen, a novel marker for hPSC^48^, showed a weak staining in all cell lines by FC, while IH analysis was negative (Fig. 1) using an antibody with validated specificity against H type 1^46^. This discrepancy between FC and IH results was noted for some antibodies and is discussed in detail below. All hPSC lines studied were negative for SSEA-1 (Table 1), known to be a marker for undifferentiated rodent cells but not found in hPSC^49^.

### HLA antigen expression in hPSC

Genomic HLA tissue typing revealed normal and expected antigen patterns of a Caucasian population (Table 1). HLA class I was abundantly expressed on all hPSC lines when analyzed by FC and IH (Figs 1 and 2B), with the exception of Val 9 that was positive in FC but negative in IH. No expression of HLA class II antigens was detected by either FC or IH.

The complement-dependent cytotoxicity crossmatch showed a high level of positive staining in all investigated cell lines, including in the negative controls. This high background staining could not be overcome despite several repetitions and protocol modifications and seemed to be caused by non-specific lysis of the hPSC upon exposure to the rabbit complement itself.

### AB(O)H histo-blood group antigen expression in hPSC

Three out of six cell lines studied were of blood group A genotype and displayed a blood group A phenotype (Fig. 1, Table 1). Interestingly, only a subpopulation of the cells (30–50%) expressed A antigens on their cell surface when analyzed by FC (Fig. 1) using three different antibody clones (Table S1). This was verified by IH (Fig. 2C, Table 1), showing clear anti-A staining of a subpopulation of cells (35–50%), while the remaining cells were completely negative. The cells were either clearly positive or completely negative and no transient antigen expression was seen. All cell lines were blood group B antigen negative.

Antibodies specific for different blood group H determinants (H type 1 and Globo H) were used to further characterize the cell lines. The Globo H structure was clearly positive for all blood group O and A cell lines using FC (Fig. 1, Table 1), and IH (Fig. 2E, Table 1). A majority of the cells in all six hPSC lines expressed blood group H type 1/SSEA-5, albeit weakly, when characterized by FC while IH was negative. The Forssman antigen (based on the globo-core saccharide), recently identified as a novel blood group system^50^, was not present in any of the analyzed cell lines.

To further characterize the expression of AB(O)H antigens in hPSC, efforts were made to distinguish between antigens carried by lipids or proteins by analyzing purified total glycosphingolipid fractions (Fig. 3A) and total protein extracts (Fig. 3B) from cell lines SA121 and SA181. Compared to the results from immunostaining of purified glycosphingolipid fractions (Fig. 3A), WB of the crude cell protein extracts gave a significant higher background (Fig. 3B). We evaluated blood group A antigen expression using three different anti-A antibodies (Table S1) at various concentrations. No specific binding of anti-A was detected in the protein extracts (data not shown), whereas the glycosphingolipid fractions revealed one single blood group A compound (Fig. 3A), which was identified as a blood group A glycosphingolipid with six sugars based on a type 1 core chain^46^. No expression of blood group B antigens was detected in the total protein extractions or the glycosphingolipid fractions (data not shown).

### Lewis histo-blood group and related antigen expression in hPSC

The type 1 core chain based Lewis blood group antigens, Le^a^ and Le^b^, were not expressed on any of the hPSC lines when analyzed by FC (Table 1) or by biochemical analysis of the SA121 and SA181 cells^46^. The type 2 chain isomers, Le^x^ and Le^y^, were both identified in all hPSC lines (Fig. 1, Table 1). Le^x^ expression followed the same pattern as for blood group A antigens, hence only a subpopulation of cells (25–65%) were positive, except for ChiPSC22 where the majority of cells expressed Le^x^ when analyzed by FC (Fig. 1). No Le^x^ expression was detected in hPSC by IH (Table 1). However, Le^y^ was intensely expressed on all cells when analyzed by both FC and IH (Figs 1 and 2F). Thin-layer chromatography immunostaining of the total neutral glycosphingolipid fractions showed several Le^y^ glycosphingolipids migrating in the hepta- to dodecaglycosylceramide region in both cell lines investigated (Fig. 3A). The anti-Le^x^ monoclonal antibody stained only slow-migrating neutral glycosphingolipids in hESC lines SA181 and SA121. Western blot analysis of the total protein extracts with anti-Le^y^ displayed one distinct band at 37kD in both hESC lines, whereas no binding was evident with anti-Le^x^ antibody (Fig. 3B).

### Sialic acid terminating antigen expression in hPSC

The sialylated type 1 core chain compound lactotetraosylceramide (sialyl-lactotetra, S-Lc_4_) was present in all hPSC lines analyzed as described above. Addition of a terminal sialic acid residue to the SSEA-1 and SSEA-3 antigens forms the S-Le^x^ and SSEA-4, respectively (Table S3). No expression of S-Le^x^ was detected in any of the analyzed hPSC lines (Table 1). All hPSC lines stained with anti-SSEA-4, were positive when analyzed by FC (Fig. 1) and IH (data not shown). Furthermore, the expression of the gangliosides GM1 and GM2 was analyzed by FC and IH in hESC line SA121 and SA181 with negative results (data not shown).

### Expression of co-stimulatory factors and CD133 in hPSC

Neither of the co-stimulatory T-cell signaling proteins B7-1 (CD80), B7-2 (CD86), ICOS (CD278) or CTLA-4 (CD152) were found by FC analysis. However, the intercellular adhesion molecule ICAM (CD54) was present on the cell surface of all analyzed cell lines (i.e. SA121, SA181, Figure S2B). The anti-CD133 antibody recognizes a carbohydrate epitope of the CD133 antigen that evidently was expressed on all hPSC lines analyzed in this study (Figure S2C).

### Alterations of HLA and histo-blood group antigen expression during differentiation

Human PSC can be stimulated *in vitro* to differentiate into cell types from all three embryonic germ layers; ectoderm, mesoderm and endoderm. In this study, one hiPSC (ChiPSC4) and three hESC (Val 9, SA121, SA181) lines were differentiated into hepatocyte-like cells (endoderm) and one hiPSC line (ChiPSC22) into cardiomyocyte-like cells (mesoderm). Flow cytometry and complementary IH analysis were performed at different time points during differentiation. In general, the experiments were repeated three times and the results were, as for the undifferentiated cells, highly reproducible within and with a few exceptions also between the investigated cell lines.

### Differentiation into hepatocyte-like cells

The alterations of antigen expression observed during differentiation into hepatocyte-like cells are shown in Figs 4 and S3. Expression of the pluripotency markers TRA-1–60, SSEA-3, SSEA-4 and S-Lc_4_ decreased during differentiation and all markers (except SSEA-4) had disappeared after 11–14 days.

HLA class I expression rapidly decreased in all cell lines (Fig. 4), with legible positivity remaining at day 7 and onwards (except ChiPSC4 that showed a slight rebound at day 21). Complementary IH showed abundant HLA class I expression on day 0 in the investigated cell lines (except Val 9, Fig. 2B, Table 1). In accordance with FC, no expression was found at day 7 or onward by IH, except for a rebound focal positivity noted exclusively in hepatocyte-like cells derived from ChiPSC4 (data not shown). HLA class II (HLA-DR/DQ/DP) antigens, absent in the pluripotent cells (see above, Table 1), did not appear during differentiation (data not shown) when analyzed by either FC or IH.

Histo-blood group AB(O)H and related carbohydrate antigens showed variable expression patterns during differentiation (Figs 4, S3). The expression of blood group H type 1/SSEA-5 and Globo H decreased, except for Val 9 that showed a rebound expression of Globo H in the hepatocyte-like cells. The blood group A positive cell lines SA121 and SA181 displayed contradicting anti-A staining patterns (Fig. 4). Complementary IH analysis of cell line SA181 showed distinct staining with anti-A on day 0 (Fig. 2C), focal traces of staining on day 4 and negative expression from day 7 and onwards (data not shown). In general, a decreasing Le^x^ and Le^y^ expression was seen by FC analysis in all cell lines (Fig. 4). Le^x^ decreased from approximately 50% positive cells to zero from day 13 and onwards. Le^y^ expression declined from 100% to about 40% positivity in all cell lines at day 21, except for hepatocyte-like SA181 cells that were Le^y^ negative. Complementary IH analysis of Le^x^ antigen demonstrated positive subpopulations of approximately 20%, 10% and 75% of cells at day 7 derived from Val 9, SA181 and ChiPSC4 respectively, whereas remaining cells were negative (Fig. 2G,H show IH images for cell line Val 9 and ChiPSC4, data for cell line SA181 are not shown). This is in accordance with the FC analysis, where the highest level of Le^x^ positive cells occurred at day 7 (range 50–80%, Fig. 4). No Le^x^ expression was detected by IH on day 0, 11 or 21 of differentiation into hepatocyte-like cells (data not shown). Immunohistochemistry demonstrated extensive anti-Le^y^ staining in all cell lines on day 0 (Fig. 2F), followed by rapidly diminishing expression during differentiation (data not shown). Sialylated Le^x^ (S-Le^x^) expression was modestly, but continuously, increased in SA121 and SA181 lines, while Val 9 and ChiPSC4 showed negligible positivity when analyzed by FC (Fig. 4). Complementary IH partly verified these results, demonstrating no expression at day 0, but a 5–10% anti-sialyl-Le^x^ positive population on day 14–21 in cell line ChiPSC4 and SA181 (Fig. 2I), but not in Val 9 (data not shown).

No expression of blood group B, Le^a^, Le^b^, or Forssman antigens was identified during the four separate time points of differentiation using FC and IH.

### Differentiation into cardiomyocyte-like cells

The pluripotency markers were eliminated during differentiation of hiPSC line ChiPSC22 into cardiomyocyte-like cells (Figs 5, S4), with the exception of SSEA-4 that showed rebound positivity. HLA class I expression during differentiation into cardiomyocyte-like cells showed a different pattern compared to the hepatocyte-like cells. Initially the expression rapidly diminished, followed by a considerable increase and all cardiomyocyte-like cells expressed HLA class I at day 26 (Fig. 5D). Similar to the hepatocyte-like cell differentiation, HLA class II antigens were not detected by FC analysis during differentiation. All cardiomyocyte-like cells were negative for blood group A, H type 1/SSEA-5, Globo H, Le^x^ and Le^y^ antigens (Fig. 5B,C). Anti-blood group B, anti-Le^a^, anti-Le^b^, anti-sialyl-Le^x^ and anti-Forssman antibodies showed no positivity in FC analysis during day 0–26 (data not shown).

### Comparison of cell surface staining of anti-SSEA-1 (MC480) and anti-Le^x^ (P12) antibodies in hPSC and their derivatives

SSEA-1 is extensively expressed in mouse but not in human PSC^32,49^ and is considered synonymous to the Le^x^ compound. However, in this study the anti-SSEA-1 antibody (clone MC480) and anti-Le^x^ antibody (clone P12) showed different staining patterns in all hPSC lines analyzed by FC (Fig. 6A). None of the cell lines showed staining with MC480 in the undifferentiated state. However, a subpopulation of the hPSC was positive for the P12 antibody (Fig. 1) and Le^x^ terminating glycosphingolipids were identified in the hPSC cells (Fig. 3A). About 10% of the cells transiently expressed SSEA-1 (median fluorescence intensity 208) on day 7 during differentiation into hepatocyte-like cells (Fig. 6B). This observation was reproducible upon experimental repetition as well as consistent between all cell lines used in this study. Complementary IH results showed a small percentage of positive cells exclusively on day 7 of differentiation in the Val 9 line, whereas the SA181 and ChiPSC4 were negative for anti-SSEA-1 throughout differentiation (data not shown). SSEA-1 was not expressed during the differentiation into cardiomyocyte-like cells (day 0, 11, 26) when analyzed by FC (data not shown).

### Alteration of CD133 expression during differentiation of hPSC

The expression of CD133 in hPSC and alterations during differentiation into hepatocyte-like and cardiomyocyte-like cells are illustrated in Figure S2C. Positive staining with anti-CD133 was found in all cell lines at day 0 during differentiation into hepatocyte-like cells, but became almost negative at day 7 to be succeeded by an increasing positivity on day 14. In contrast, during differentiation of ChiPSC22 into cardiomyocyte-like cells the expression of CD133 declined rapidly and was undetectable from day 11 and onwards.

## Discussion

The HLA and ABO histo-blood group systems are the major immune barriers in organ transplantation. The initial perception of an immune privileged state in hPSC has been challenged in several studies^9,10^. In contrast to previous studies^12,13^, all six hPSC lines in our study extensively expressed cell surface HLA class I antigens, which are potential targets for the immune system of a HLA-mismatched recipient. The HLA class I expression diminished during differentiation into hepatocyte-like cells (except for one cell line), in congruence with the adult status. Unexpectedly, differentiation into cardiomyocyte-like cells resulted in a rebound expression of HLA class I at day 21 although adult cardiomyocytes lack HLA class I antigens^17^. This, as well as other antigen fluctuations noted during differentiation could be a consequence of different developmental processes but also a potential rebound effect driven by culturing conditions and differentiation protocols. Identification of such external factors altering immunogenicity could facilitate manipulation of differentiation into products with more favorable antigenicity and must be further investigated before therapeutic hPSC-based applications can be realized. The HLA-genotyping of all cell lines disclosed expression of alleles in consistency with their presumed origin from a Caucasian population and in alignment with earlier studies^15,51^. Therefore, as in allotransplantation, it is necessary to establish the HLA haplotypes of the donor and recipient in stem cell therapy. Our efforts to investigate the functional significance of the expressed HLA antigens were not successful due to a high non-specific background, most likely caused by an unspecific lysis of the hPSC by the rabbit complement itself.

Favorable transplantation aspects are that, in analogue with earlier studies^12,15,51,52^, no HLA class II antigens were found on the hPSC. Furthermore, the hPSC did not express co-stimulatory factors (i.e. B7-1/CD80, B7-2/CD86, ICOS/CD278, CTLA-4/CD152) necessary for successful T-lymphocyte activation.

Initially it was unclear if the antigens of the blood group ABO system were expressed on hPSC^28^. However, IH studies of nine hESC lines revealed blood group A and B antigen expression^29^ which was confirmed by biochemical analyses^46^. In the SA121 and SA181 lines, both blood group A and H glycosphingolipids based on a type 1 core saccharide chain (lactotetra) were identified, while the Le^a^/Le^b^ compounds were lacking^46^. Since the *Le* gene frequency in the Caucasian population is approximately 80%^53^ it is likely that some of the hPSC lines should be *Le* positive. This indicates that the *Le* gene coded enzyme is not active in hPSC. The opposite relationship was seen for the expression of corresponding blood group antigens based on type 2 (neo-lactotetra) core saccharides. No type 2 chain based A antigens were found while the Le^x^/Le^y^ compounds were present showing that the fucosyltransferase working on the neolactotetra saccharide substrate was active. Interestingly, a subpopulation of 30–45% of the hPSC cells within a specific cell line expressed blood group A antigen, whereas the majority were completely negative when analyzed by either FC or IH. This may be a consequence of the blood group A_1_ glycosyltransferase becoming active at certain stages of cell development and may reflect a heterogeneous cell population in various stages of maturation. Furthermore, in contrast to adult human organs/cells that contain several structurally different blood group A glycosphingolipids, cell lines SA121 and SA181 only contained one blood group A glycosphingolipid compound with six sugar residues^29^. No blood group A antigens carried by proteins were found in the blood group A genotype cell lines, suggesting that A antigens are mainly lipid-linked in hPSC. This is in contrast to adult human erythrocytes were a majority of AB(O)H antigens are linked to proteins^53^ and the AB(O)H glycosphingolipids are based on type 2 core chains^54^. During differentiation into cardiomyocyte-like cells, expression of blood group A antigens diminished and was negligible from day 11, which is concordant with adult cardiomyocytes lacking A antigens^30^. However, the alterations during differentiation into hepatocyte-like cells were more inconsistent, with decreasing expression in cell line SA181 in accordance with adult hepatocytes lacking AB(O)H antigens and the study by Mölne *et al*. demonstrating retained blood group B antigen expression in the hESC-derived hepatocyte-like cells^27,29,31^. In contrast, A antigen expression was increased in the SA121 line.

The use of several different analytical techniques strengthened our results regarding the detection and expression of antigen epitopes in the cell lines. Nonetheless, there are methodological aspects to be discussed depending on the analytical techniques used. Generally, we found weaker staining in IH compared to FC analyses. However, with few exceptions, the results were congruent between the two antibody-dependent methods. For example, no expression of Le^x^ could be detected by IH in the hPSC, but positive staining was obtained during differentiation in alignment with more intense expression in FC analysis. The discrepancies may be due to the higher sensitivity of FC or alterations in epitope-structures caused by the fixation and deparaffinization processes used in IH^55^. Furthermore, biochemical analysis of the cell lines available in sufficient amounts strengthened the identification of the carbohydrate antigens identified by the immune techniques.

Phenotypic profiling with immunostaining techniques is an essential tool to evaluate pluripotency in stem cells. The different techniques and markers used in this study, together with alteration of the markers during cell differentiation, enabled us to compare presumed pluripotency markers. Compared to TRA-1–60 and sialyl-lactotetra that showed an anticipated and technique-independent expression pattern, SSEA-3 and SSEA-4 were more suboptimal markers. Anti-SSEA-3 showed a diverse and weak staining of hPSC in FC analysis, which has been previously described^1^, and was partly explained by its mainly intracellular localization reveled by IH. On the contrary, SSEA-4 was abundantly expressed in the hPSC with a remaining positivity in 15% of the hepatocyte-like cells consistent with earlier work^56^. In the cardiomyocyte-like cells an increased expression from day 11 and onwards was found congruent with findings that adult cardiomyocytes express SSEA-4^57^. The H type 1/SSEA-5 antigen has recently been proposed to be a marker of undifferentiated cells^48^. This is partly confirmed by our study showing that H type 1/SSEA-5 expression in hPSC diminishes upon differentiation. However, the level of antigens and fluorescence intensity in the pluripotent cells was low as seen in the FC histograms, and no reaction was found with IH analysis.

The results from this study, together with our previous work^47,58^, shows that sialyl-lactotetra (s-Lc_4_) can be used as a phenotypic pluripotency marker. Based on this work this may also be the case for the Le^y^ epitope. Both antibodies work well in various immunostaining techniques and can besides being markers of undifferentiated cells, be used as selection tools during differentiation for exclusion of undifferentiated cells in heterogeneous cultures. These two structurally well-defined antigens are lost upon differentiation into hepatocyte-like (i.e. endoderm), cardiomyocyte-like (i.e. mesoderm) and neural stem cells (i.e. ectoderm,^58^). However, in contrast to sialyl-lactotetra, the Le^y^ antigen is present in some adult human tissues and in a fraction of hepatocyte-like and neural stem cells, but not in cardiomyocyte-like cells. Noteworthy, functional pluripotency assays using anti-S-Lc_4_ or anti-Le^y^ have not yet been done.

Another stage-specific embryonic antigen of interest is SSEA-1, abundantly expressed in mouse but not in human PSC^32,49^. SSEA-1 is often presented as synonymous to CD15 and Le^x^, which is a misconception due to the fact that the antibody defining SSEA-1 (clone MC480^32^) is specific for the Le^x^ epitope exclusively when presented on extended type 2 core chains^59,60^. This specificity entails the presence of Le^x^, but not SSEA-1, on hPSC found in this study. None of the cell lines expressed SSEA-1 in the pluripotent state, but about 10% of the cell population showed transient positivity on day 7 of differentiation into hepatocyte-like cells. This pattern is consistent with earlier work on embryonal carcinoma cells differentiated by retinoic acids, noting a peak of SSEA-1 expression on day 7 followed by declining expression^61^.

The transmembrane glycoprotein CD133 is often used to select or identify stem cells, such as hematopoietic stem cells and so-called cancer stem cells. This role has been challenged in recent years^62^. In this study, we found a fluctuating expression of CD133 during the differentiation into hepatocyte-like cells, while the expression diminished during differentiation into cardiomyocyte-like cells. The dynamic expression in differentiated, but still immature, cells contradicts the usability of CD133 as a marker for, and a target for drug development against, cancer stem cells.

The biological equivalence of hESC and hiPSC has been vividly debated^63^. We have previously shown that the composition of glycosphingolipids are similar between the two hPSC types, with the exception of reduced levels of GD1a, GD1b and increased levels of GM3 that was found in hiPSC compared to hESC^58^. In this study, we compared three hESC with three hiPSC lines and their derivatives and could not detect any apparent systematic differences in expression of HLA or AB(O)H histo-blood group antigens. However, our results indicate cell line specific expression patterns of tissue antigens in the pluripotent and differentiated states that are not generalizable to all hPSC lines. Consequently, all cell lines most be characterized individually before selecting suitable lines for clinical applications.

The results obtained in this descriptive analytical study, in addition to be a basic scientific exploration, also have clear clinical implications in the further development of the stem cell field with the ultimate goal to be used in the clinic for treatment of patients with end stage cell/organ failure. In conclusion, this study clearly demonstrates the necessity for hPSC-based therapies to address the status of the HLA and ABO blood group systems before clinical use.

## Electronic supplementary material

Supplemental Material
