# Supplementary material for: HLA and Histo-Blood Group Antigen Expression in Human Pluripotent Stem Cells and their Derivatives
Source: Sci Rep. 2017 Oct 12;7:13072. doi: 10.1038/s41598-017-12231-8 (PMC5638960; doi:10.1038/s41598-017-12231-8)
Supplement: Supplementary file 1 — Supplemental Material [file 41598_2017_12231_MOESM1_ESM.pdf]

HLA and Histo-Blood Group Antigen Expression in Human Pluripotent Stem Cells and their Derivatives

Karin Säljö[1], Angela Barone[2], Johan Mölne[3], Lennart Rydberg[4], Susann Teneberg[2] and Michael E Breimer[1]

[1] Department of Surgery, Institute of Clinical Sciences, Sahlgrenska Academy at University of Gothenburg, Sahlgrenska University Hospital, Sweden.

[2] Department of Medical Biochemistry and Cell Biology, Institute of Biomedicine, Sahlgrenska Academy at University of Gothenburg, Sweden

[3] Department of Pathology and Genetics, Institute of Biomedicine, Sahlgrenska Academy at University of Gothenburg, Sahlgrenska University Hospital, Sweden

[4] Department of Clinical Immunology and Transfusion Medicine, Institute of Biomedicine, Sahlgrenska Academy at University of Gothenburg, Sahlgrenska University Hospital, Sweden

Key words: Human pluripotent stem cells, Cell surface antigens, HLA, ABO blood group system, Differentiation, Hepatocyte-like cells, Cardiomyocyte-like cells

Corresponding author: Karin Säljö, MD, Sahlgrenska University Hospital, S-41346 Gothenburg, Sweden, +46313427624, E-mail: karin.saljo@vgregion.se

SUPPLEMENTAL MATERIAL

Flow cytometry analysis

In general, the experiments were repeated three times with a minimum interval of one week. Duplicate samples were prepared each time to account for day-to-day fluctuations and sample preparation variations. Single cell suspensions of hiPSC and hESC lines were obtained by incubation with TrypLE™ Select (Invitrogen) at 37°C. Following repeated washing in medium or phosphate buffered saline (PBS) the cells were passed through a 40µm cell strainer (Becton Dickinson), stored and stained at 4°C. A cell suspension containing 2-3x10^5 cells was distributed in each tube and centrifuged for 5 min at 260 or 400g (differentiated and undifferentiated cells, respectively), followed by extraction of the supernatant by Vacusafe aspiration system (Becton Dickinson). This procedure was repeated for each washing or staining step. Subsequently, cell pellets were incubated with 25µL of primary antibody and corresponding isotype control (see Supplemental Table S1 for details) for 30 minutes and thereafter washed three times in 4°C phosphate buffered saline with fetal calf serum (PBS-FCS, used as washing-solution throughout the protocol). Cells stained with unconjugated antibodies were thereafter incubated with FITC-conjungated secondary antibodies (see Supplemental Table S2 for details) for 30 min. After washing, the final cell pellet was resuspended in 200µl PBS and analyzed using a FACSCalibur™ flow cytometer (Becton Dickinson). In general, 20 000 fluorescence signals were acquired per sample using the CellQuest™ software (Becton Dickinson) on a logarithmic scale and analyzed using the FlowJo (v10.1.r5) software. To exclude dead cells and debris, propidium iodide exclusion (0.5 µg/ml; Sigma Chemicals Co) was performed and the forward and side light scatters were adjusted accordingly (see representative illustration in Supplemental Figure S1).
The flow cytometry results are mainly presented and evaluated as percentage of positive cells instead of values of mean fluorescence intensity (MFI), which is a strategy that has been postulated to be more relevant when assessing pluripotency [1]. To calculate the percentage of positive cells expressing the antigen of interest the threshold was set at the point of the histogram where 99% of the cells in the negative control were indeed interpreted as negative. No significant difference in fluorescence was detected between cells stained with corresponding isotype control or secondary antibody alone indicating modest non-specific binding. Therefore, the negative control used for defining the negative gates consisted of cells incubated only with relevant secondary antibodies unless otherwise stated throughout the experiments.

Supplemental Figures

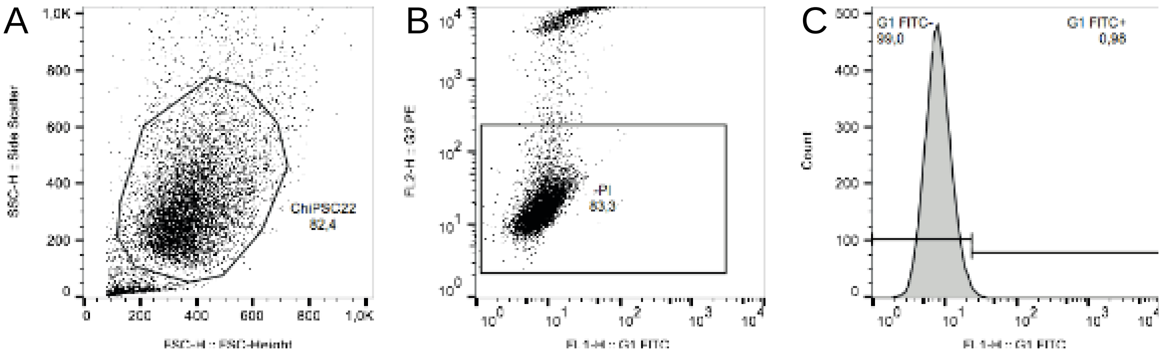

Supplemental Figure S1. Illustration of the gating procedures used in flow cytometry analysis. A representative flow cytometry analysis (**A**), propidium iodide exclusion method (**B**) and gating-procedure (**C**) used in this study exemplified by the negative control sample of induced pluripotent stem cell line ChiPSC22. Dead cells and debris were excluded by adjusting forward and side light scatters (**A**) and staining with propidium iodide (**B**). Chart **C** illustrates the procedure used for defining the cut-off value, set when 99% of the negative control sample falls within the negative gate, hence separating negative and positive cells.

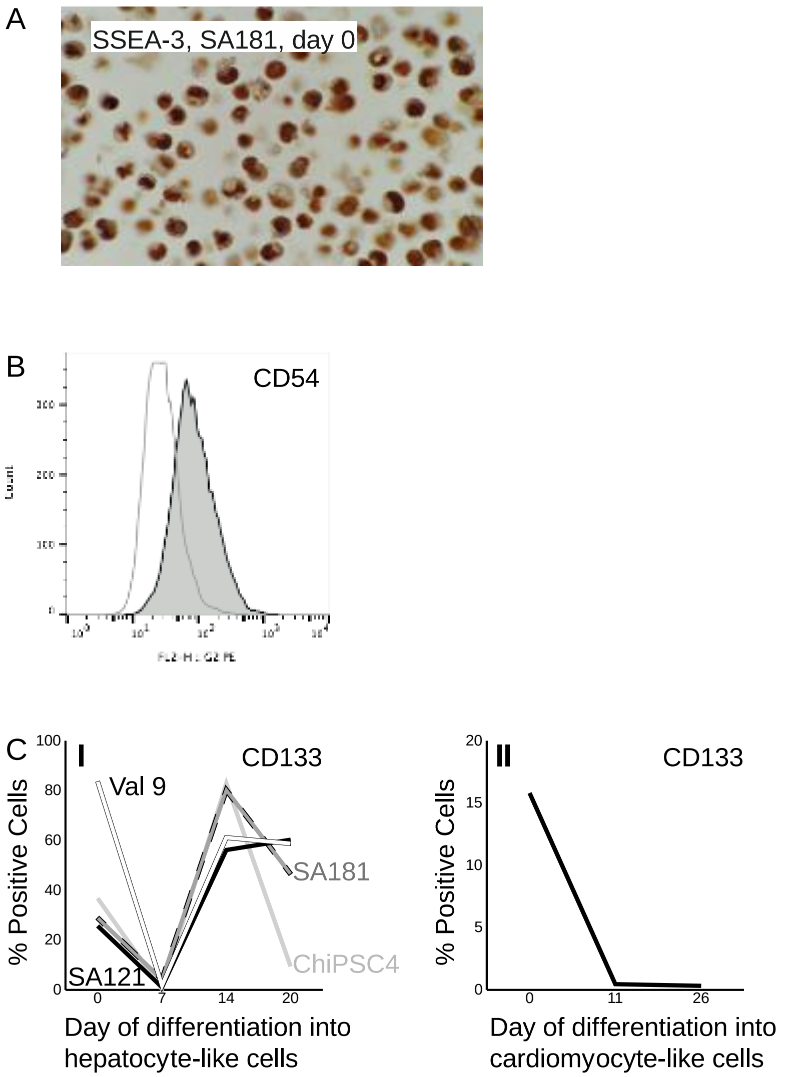

Supplemental Figure S2.
A. Immunohistochemical detection of SSEA-3 in the human embryonic stem cell line SA121. SSEA-3 is mainly expressed in the intracellular compartments but also weakly expressed on the cell surface.
B. Expression of ICAM (CD54) on the cell surface of human embryonic stem cells. The histogram shows a representative expression of ICAM (CD54) on the cell surface of cell line SA121 analyzed by flow cytometry. The filled grey histogram illustrates SA121 cells incubated with FITC-conjugated anti-ICAM (CD54) antibody and the transparent grey curve is a negative control, consisting of the corresponding isotype control.
C. Alterations of CD133 expression during differentiation into hepatocyte- (**I**) and cardiomyocyte-like cells (**II**). The expression of CD133 fluctuated, although rather coherently between the different cell lines, during differentiation into hepatocyte-like cells (**I**). The expression of CD133 rapidly declined during differentiation of cell line ChiPSC22 into cardiomyocyte-like cells and was not detectable by flow cytometry from day 11 and onwards (**II**).

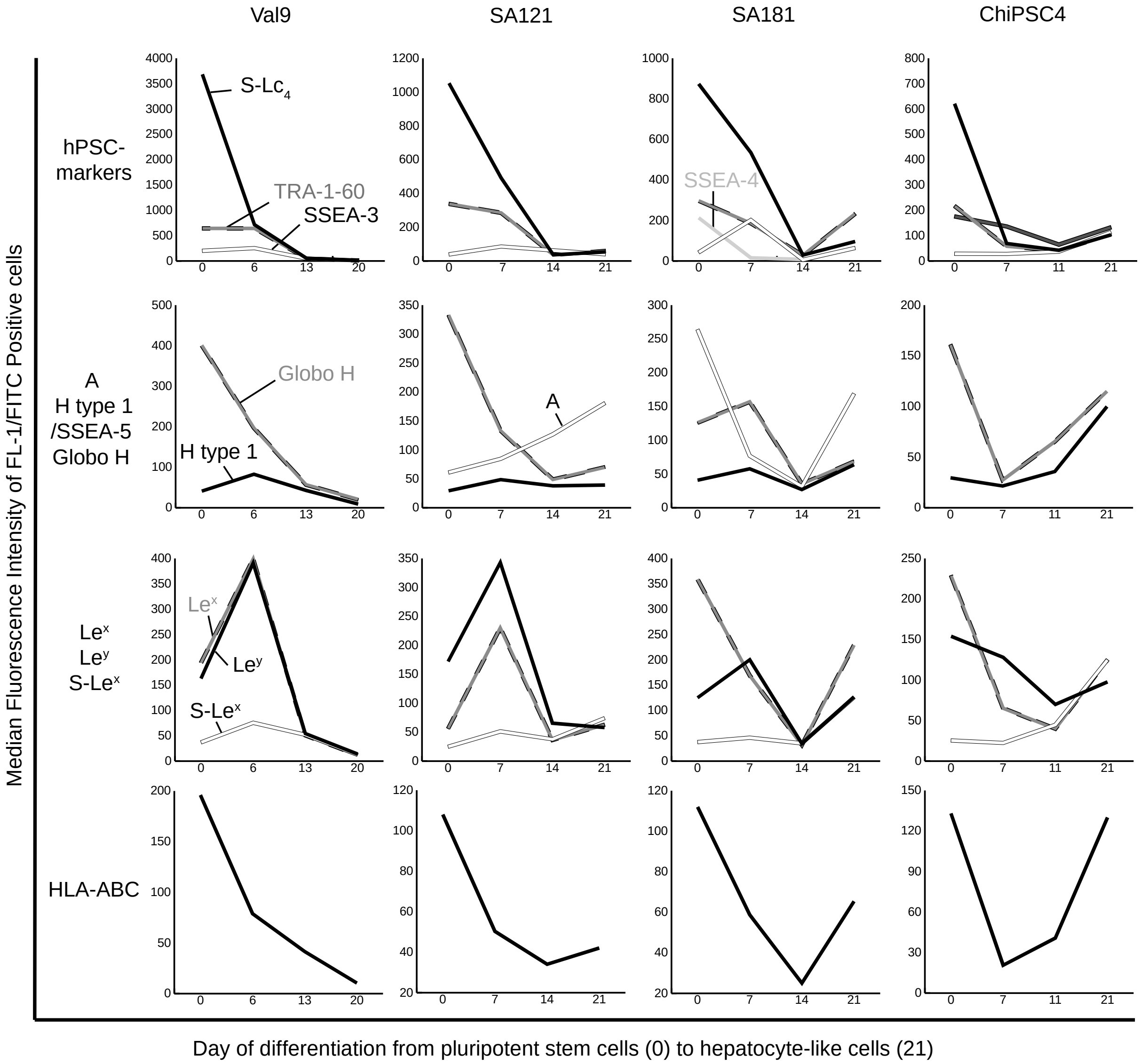

Supplemental Figure S3. Alteration of median fluorescence intensity of HLA and histo-blood group antigen expression during differentiation of human pluripotent stem cells into hepatocyte-like cells.

The human embryonic stem cell lines Val9, SA121 and SA181, and the human induced pluripotent stem cell line ChiPSC4 were characterized by flow cytometry with monoclonal antibodies. The figure presents the median fluorescence intensity (MFI) of the FL-1/FITC positive cells from one representative analysis. *Abbreviations; hPSC, human pluripotent stem cells; S-Lc4, sialyl-lactotetra; A, blood group A; Le^x, Lewis x; Le^y, Lewis y; S-Le^x, sialyl-lewis x.*

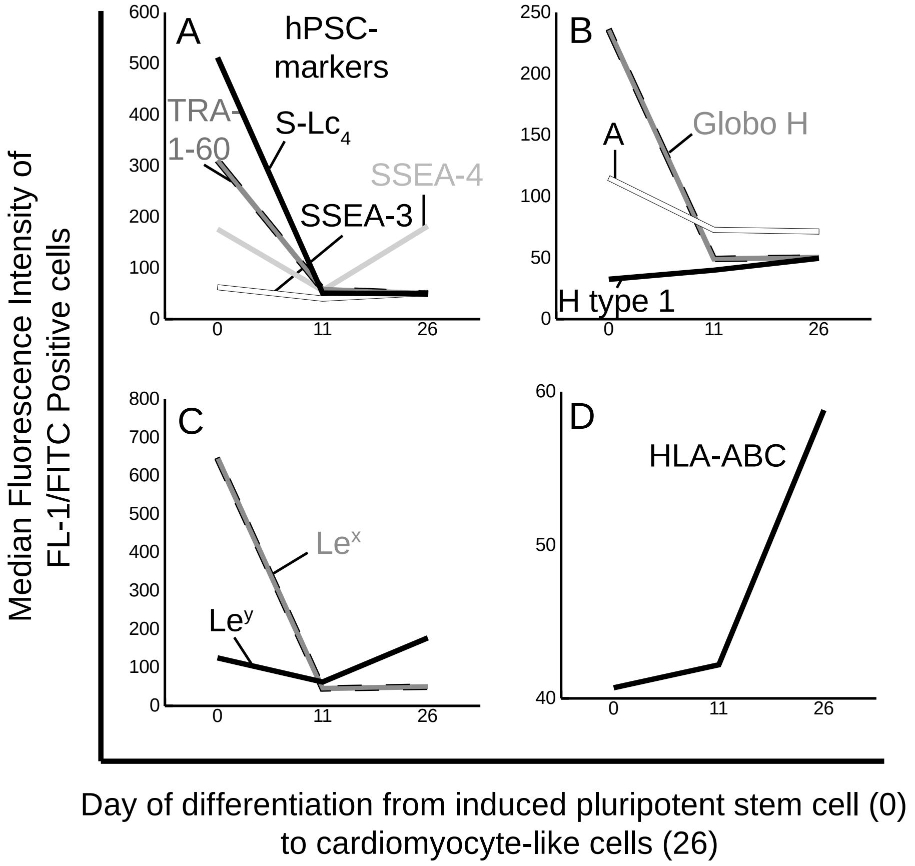

Supplemental Figure S4. Alteration of median fluorescence intensity of HLA and histo-blood group antigen expression during differentiation of human induced pluripotent stem cell line ChiPSC22 into cardiomyocyte-like cells.
The human induced pluripotent stem cell line ChiPSC22 was differentiated into cardiomyocyte-like cells and the median fluorescence intensity (MFI) of different HLA and histo-blood group antigens was analyzed. The figure presents MFI of FL-1/FITC positive cells from one representative analysis. *Abbreviations; hPSC, human pluripotent stem cells; S-Lc$_4$, sialyl-lactotetra; A, blood group A; Le^x, Lewis x; Le^y, Lewis y; S-Le^x, sialyl-lewis x.*

Supplemental Tables

Primary Antibodies	Iso-type	Clone	Cat. No.	Manufacturer	Dilutions FC	IH	CBA	WB
Anti-blood group A	IgM	HE-193	ab2521	Abcam, ref 2	1:50	1:50	1:500	1:1000
Anti-blood group A	IgM	AO581	n.p.	Dakopatts ref 2	1:50			1:100
Anti-blood group A	IgM	Z2B-1	sc-52367	Santa Cruz	1:50			1:100
Anti-blood group B	IgM	HEB-29	ab2524	Abcam, ref 2	1:50	1:100	1:100	1:100
Anti-H type 1/ SSEA-5	IgG3	17-206	ab3355	GeneTex/ Abcam, ref 3	1:50	1:50		
Anti-Le^a	IgG	T174	ab3356	Abcam	1:50			
Anti-Le^b	IgM	T218	ab3357	Abcam	1:50			
Anti-Le^x	IgM	P12	434631	Calbiochem	1:10	1:50	1:200	
Anti-Le^x	IgM	P12	ab3358	Abcam	1:10	1:200		1:100
Anti-Le^y	IgM	F3	434636	Calbiochem	1:10	1:50	1:200	
Anti-Le^y	IgM	F3	ab3359	Abcam	1:10	1:200		1:100
Anti-Sialyl-Le^x	IgM	KM93	565953	Calbiochem	1:10	1:25		
Anti-SSEA-1	IgM	MC-480	ab16285	Abcam	1:100	1:100		
Anti-SSEA-3	IgM	MC-631	148833	eBioscience	1:100	1:100		
Anti-SSEA-4	IgG3	MC813-70	148843	eBioscience	1:50	1:500		
Anti-Globo-H	IgM	Mbr1	ALX804 550	Enzo Life Science	1:100	1:50		
Anti-Sialyl lactotetra	IgM	TRA4	n.c.	Svennerholm et al, ref 4	1:100	1:500		
Anti-Forssman	IgM	E28-4/1825	n.c.	Kind gift from Lola Svensson	1:20			
Anti-GM1	IgM	GM1:1	n.c.	Molander et al ref 5	1:10			
Anti-GM2	IgM	DMAb-1	n.c.	Karlsson et al ref 6	1:5			
Anti-TRA-1-60	IgM	TRA-1-60	ab16288	Abcam	1:100	1:100		
Anti-HLA-ABC-FITC conjugated	IgG2a	W6/32	11-9983-41	eBioscience	1:20			
Anti-HLA-ABC	IgG2a	W6/32	ab95821	Abcam		1:50		
Anti-HLA-DQ	IgG2a		ab55158	Abcam	1:20	1:100		
Anti-HLA-DP	IgG		ab88089	Abcam	1:20	1:100		
Anti-HLA-DR	IgG2a	L243	ab136320	Abcam	1:50	1:100		
Anti-CD54/ICAM	IgG1	HA58	12-0549	eBioscience	1:10			

Anti-CD80/B7-1	IgG1	2D10.4	11-0809	eBioscience	1:10	
Anti-CD86/B7-2	IgG2b	IT2.2	12-0869-71	eBioscience	1:10	
Anti-CD133	IgG1	AC133	130-090-422	Miltenyli Biotec	1:10	1:25
Anti-CD152/ CTLA-4	IgG2a	14D3	12-1529-71	eBioscience	1:10	
Anti-CD278/ICOS	IgG1	ISA-3	11-9948	eBioscience	1:10	

Supplemental Table S1. Summary of primary antibodies used in this study.
All antibodies used in this study were raised in mouse except for the SSEA-3 and Forssman antibodies that were of rat origin.
Abbreviation: n.c., not commercially available; n.p, no longer in production; FC, flow cytometry; IH, immunohistochemistry; CBA, chromatogram binding assays; WB, western blot.

Secondary Antibodies and Isotype Controls	Cat. No.	Manufacturer	Dilutions		
			FC	IH	WB
FITC conjungated Anti-Mouse IgG	11-4011-85	eBioscience	1:100		
FITC conjungated Anti-Mouse IgM	sc-2082	Santa Cruz	1:100		
FITC conjungated Anti-rat IgM	110990	eBioscience	1:200		
Alkaline phosphatase conjungated Anti Mouse Ig	1010-04	Southern Biotech			1:3000
Biotin conjungated Anti-Rat IgM	ab97178	Abcam		1:200	
Biotin conjungated Anti-Mouse IgM+IgG	ab47844	Abcam		1:200	
Biotin conjungated Anti-Rabbit IgG	pk6101	Vector laboratories		1:200	
Biotin conjungated Anti-Mouse IgG	pk6102	Vector laboratories		1:200	
HRP conjungated Anti-Mouse IgM	Ab77230	Abcam		1:200	
DAKO EnVision®+System-HRP, Anti-Mouse	K4007	Dako		a.k.	
Isotype Control mouse IgG (FITC)	ab37356	Abcam	1:100		
Isotype Control mouse IgM (FITC)	ab91546	Abcam	1:200		
Isotype Control rat IgM (FITC)	ab35774	Abcam	1:200		

Supplemental Table S2. Summary of secondary antibodies and isotype controls used in this study. *Abbreviations: FC, flow cytometry; IH, immunohistochemistry; WB, western blot.*

Trivial name	Antigen determinant
H type 1/SSEA5	Fucα2Galβ3GlcNAcβ3-R
Globopenta/SSEA-3	Galβ3GalNAcβ3Galα4Galβ4Glcβ1Cer
Globo H	Fucα2Galβ3GalNAcβ3Galα4Galβ4Glcβ1Cer
Sialyl-globopenta/SSEA-4	NeuAcα3Galβ3GalNAcβ3Galα4Galβ4Glcβ1Cer
Le^a	Galβ3(Fucα4)GlcNAcβ3-R
Le^b	Fucα2Galβ3(Fucα4)GlcNAcβ-R
Le^x/SSEA-1	Galβ4(Fucα3)GlcNAcβ-R
Sialyl-Le^x	NeuAcα3Galβ4(Fucα3)GlcNAcβ-R
Le^y	Fucα2Galβ4(Fucα3)GlcNAcβ-R
Blood group A antigen	GalNAcα3(Fucα2)Galβ-R
Blood group B antigen	Galα3(Fucα2)Galβ-R
Sialyl-lactotetra	NeuAcα3Galβ3GlcNAcβ-R
Forssman	GalNAcα3GalNAcβ3Galα4Galβ4Glcβ1Cer

Supplemental Table S3. Chemical structures and shorthand designations of carbohydrate histo-blood group antigens studied.

SUPPLEMENTAL REFERENCES

1 Ramirez, J. M. *et al.* Brief report: benchmarking human pluripotent stem cell markers during differentiation into the three germ layers unveils a striking heterogeneity: all markers are not equal. *Stem Cells* **29**, 1469-1474 (2011).

2 Svennerholm, L. *et al.* Human brain gangliosides: developmental changes from early fetal stage to advanced age. *Biochimica et Biophysica Acta (BBA)-Lipids and Lipid Metabolism* **1005**, 109-117 (1989).

3 Molander, M., Berthold, C.-H., Persson, H., Andersson, K. & Fredman, P. Monosialoganglioside (GM1) immunofluorescence in rat spinal roots studied with a monoclonal antibody. *J. Neurocytol.* **26**, 101-111 (1997).

4 Karlsson, G., Månsson, J.-E., Wikstrand, C., Bigner, D. & Svennerholm, L. Characterization of the binding epitope of the monoclonal antibody DMAb-1 to ganglioside GM2. *Biochimica et Biophysica Acta (BBA)-Lipids and Lipid Metabolism* **1043**, 267-272 (1990).
